
JOURNAL INFORMATION
==============================
NLM Title Abbreviation: J Cell Mol Med
Journal ID: J. Cell. Mol. Med
NLM Journal ID: 101083777
Journal ID: jcmm

Journal of Cellular and Molecular Medicine

ISSN: 1582-1838
EISSN: 1582-4934
Publisher: Wiley

ARTICLE INFORMATION
==============================
PMCID: PMC3822583
DOI: 10.1111/j.1582-4934.2013.01635.x
Article version: 1
Subjects: Issue Information

Issue Information

Print publication date: 2013 Feb
Electronic publication date: 2013 Feb 27
Volume: 17
Issue: 2
First page: i
Last page: i
Copyright: Copyright © 2013 Foundation for Cellular and Molecular Medicine/Blackwell Publishing Ltd.
Copyright year: 2013
License: Re-use of this article is permitted in accordance with the Creative Commons Deed, Attribution 2.5, which does not permit commercial exploitation.
License URL: https://creativecommons.org/licenses/by/2.5/

==============================
### Journal of Cellular and Molecular Medicine

5-year Impact Factor: 5.818

### Editor-in-Chief: L.M. Popescu

Head, Department of Cellular and Molecular Medicine, Faculty of Medicine
'Carol Davila' University of Medicine and Pharmacy, Bucharest, Romania
Tel: +40 744 535 298; Fax: +40 21 312 4885; E-mail: LMP@jcmm.org

### Editorial Board

**P. Anversa,** Boston, USA
**J.J.M. Bergeron,** Montreal, Canada
**M. Bhatia,** Christchurch, New Zealand
**O. Binah,** Haifa, Israel
**H.E. Blum,** Freiburg, Germany
**T.B. Bolton,** London, UK
**C. Bona,** New York, USA
**M.D. Bootman,** Cambridge, UK
**N. Bornstein,** Tel Aviv, Israel
**G. Bussolati,** Turin, Italy
**K. Camphausen,** Bethesda, USA
**M. Caraglia,** Naples, Italy
**Y.H. Chen,** Shanghai, China
**S.N. Constantinescu,** Brussels, Belgium
**P. Doevendans,** Utrecht, The Netherlands
**B. Eyden,** Manchester, UK
**M.S. Faussone-Pellegrini,** Florence, Italy
**W. Franke,** Heidelberg, Germany
**L. Frati,** Rome, Italy
**T. Fujimoto,** Nagoya, Japan
**P.M. Glazer,** New Haven, USA
**J. Gooch,** Atlanta, USA
**S.K. Gupta,** New Delhi, India
**H. zur Hausen,** Heidelberg, Germany
**A.M. Hofer,** Boston, USA
**R.E. Horch,** Erlangen, Germany
**O. Hovatta,** Huddinge, Sweden
**S.S. Hu,** Beijing, China
**J.D. Huizinga,** Hamilton, Canada
**M. Ivan,** Indianapolis, USA
**B. Jena,** Detroit, USA
**Y.T. Konttinen,** Helsinki, Finland
**T. Kornberg,** San Francisco, USA
**S. Kostin,** Bad Nauheim, Germany
**R.C. Kukreja,** Richmond, VA, USA
**R. Langer,** Cambridge, USA
**J.W. Lawler,** Boston, USA
**F. Lupu,** Oklahoma City, USA
**J. Meldolesi,** Milan, Italy
**M. Mercola,** San Diego, USA
**V.M. Miller,** Rochester, USA
**K. Morgan,** Boston, USA
**J.F. Morris,** Oxford, UK
**C. Mummery,** Utrecht, The Netherlands
**F. Murad,** Houston, USA
**D.F. Mureşanu,** Cluj-Napoca, Romania
**H. O'Neil,** Canberra, Australia
**K.P. Nephew,** Bloomington, USA
**M. Pesce,** Milan, Italy
**O.H. Petersen,** Liverpool, UK
**N.C. Popescu,** Bethesda, USA
**D. Pozo,** Sevilla, Spain
**M.Z. Ratajczak,** Louisville, USA
**U. Ripamonti,** Johannesburg, South Africa
**J. Rubin,** Bethesda, USA
**A. Samali,** Galway, Ireland
**P.R. Sanberg,** Tampa, USA
**R.C. dos Santos Goldenberg** Rio de Janeiro, Brasil
**M. Simionescu,** Bucharest, Romania
**G.W. Sledge,** Indiana, USA
**R.V. Stan,** Hanover, USA
**G. Steinhoff,** Rostock, Germany
**M. Stürzl,** Erlangen, Germany
**M. Taggart,** Newcastle upon Tyne, UK
**A. Tosaki,** Debrecen, Hungary
**N.A. Turner,** Leeds, UK
**C.A. Vacanti,** Boston, USA
**A. Vaheri,** Helsinki, Finland
**L. Vécsei,** Szeged, Hungary
**V. Veloulescu,** Baltimore, USA
**C. Wang,** Beijing, China
**R.A. Wang,** Xi'an, China
**B. Winblad,** Stockholm, Sweden
**C. Zhang,** Newark, USA

### Executive Assistant Editor:

**M. Leabu,** Bucharest, Romania

### Executive Assistant Editor:

Asia Office
**X. Wang,** Shanghai, China

### Assistant Editors:

**H. Amin,** Houston, USA
**K. Cheng,** Los Angeles, CA, USA
**L. Chirieac,** Boston, USA
**Sanda M. Ciontea**, Bucharest, Romania
**V. Cismasiu,** Oxford, UK
**Camelia Iancu-Rubin,** New York, USA
**Elisa-Anamaria Liehn,** Aachen, Germany
**N.I. Moldovan,** Columbus, USA
**M. Oprica,** Stockholm, Sweden
**A.G. Papavassiliou,** Athens, Greece
**R.A. Towner,** Oklahoma City, USA

### Publishing Editors:

**D. Cretoiu,** Bucharest, Romania
**C.G. Manole,** Bucharest, Romania

### Consultant Editor:

**M.E. Hinescu,** Bucharest, Romania

### Editorial Office

*Journal of Cellular and Molecular Medicine*, P.O. Box 35-29, Bucharest 35, Romania.
Tel./Fax: +40 21 312 4885. E-mail: editors@jcmm.org

### Publisher

*Journal of Cellular and Molecular Medicine*, Blackwell Publishing Ltd, 9600 Garsington Road, Oxford, OX4 2DQ UK.
Tel.: +44 1865 776868, Fax: +44 1865 714591.
Production Editor: Jeremy Chua (jcmm@wiley.com)

Copyright © 2013 Foundation for Cellular and Molecular Medicine/Blackwell Publishing Ltd.

INDEXED in: Index Medicus - Medline - Current Contents - Science Citation Index...

Journal of Cellular and Molecular Medicine
(Online) ISSN 1582-4934
